# Asymbiotic in vitro seed germination, in vitro seedling development, and ex vitro acclimatization of *Spiranthes*

**DOI:** 10.1002/aps3.11494

**Published:** 2022-10-01

**Authors:** Peter J. Zale, Ashley Clayton, John Nix, Matt Taylor

**Affiliations:** ^1^ Research and Conservation Division of Horticulture, Longwood Gardens P.O. Box 501 Kennett Square Pennsylvania 19348 USA; ^2^ Department of Horticultural Science North Carolina State University 2721 Founders Drive Raleigh North Carolina 27607 USA; ^3^ Curio Wellness 215 Washington Avenue, Suite 600 Towson Maryland 21204 USA

**Keywords:** asymbiotic, exceptional species, in vitro, Orchidaceae, seeds, *Spiranthes*

## Abstract

**Premise:**

Reproducible seed propagation and production protocols were developed for *Spiranthes* and related taxa to facilitate ex situ conservation practices.

**Methods and Results:**

*Spiranthes* seeds were scarified for 3‐ and 10‐min intervals in 10% sodium hypochlorite solution, then cultured on three seed germination media. After germination, seedlings were given one of the three photoperiod treatments, and then planted in one of four greenhouse substrates. Seed germination ranged from 0% to 90% and occurred on all three media only after the 3‐min scarification. Seedlings in the 24/0‐h light/dark and 16/8‐h light/dark photoperiods on P723 medium had significantly higher fresh weight than those in the dark treatment group. Ex vitro survival ranged from 55% to 95% across substrates.

**Conclusions:**

Results show that *Spiranthes* seeds are damaged by extended chemical scarification, are adaptable to a variety of culture media, and require light for optimal development. Further experimentation showed that the propagation protocols described here can be applied broadly within the genus.

Routine, in vitro seed propagation and seedling production remains elusive for many genera of North American native orchids. In vitro propagation efforts are affected by unknown seed germination scarification and media requirements, the role of light in the continued development of in vitro seedlings, and substrate requirements for acclimatization to ex vitro conditions (Zettler and McInnis, 1993; Malmgren, 1996; Stewart and Kane, 2007; Kauth et al., 2008). Although significant advances made with certain genera, such as *Cypripedium* L., have resulted in successful large‐scale horticultural production and in situ/ex situ conservation efforts, information related to many genera, including *Spiranthes* Rich., is scattered or remains unpublished (Steele, 1996; Seaton et al., 2013). Like other orchids, *Spiranthes* species can be considered “exceptional” (i.e., they cannot be conserved long‐term using conventional seed banking methods or require special handling and conditions for germination) due to the laboratory requirements for propagation of the dust‐like seeds, unknown seed desiccation tolerance and lifespan, and variable natural seed production in some species resulting from a variety of factors (Reddoch and Reddoch, 2009; Pence et al., 2022). Therefore, studies that result in baseline, reproducible propagation protocols for *Spiranthes* would be of use to horticulturists, conservationists, restoration biologists, seed scientists, citizen scientists, and others interested in applied orchid and exceptional species conservation.

*Spiranthes*, known commonly as ladies’‐tresses orchids, is a widespread genus containing ca. 45 species found throughout North and South America, Eurasia, and Australia (Catling, 1980; Sheviak and Brown, 2002). Eastern North America is the center of diversity for the genus with ~23 species, where they occupy a wide variety of ecosystem and habitat types and serve as biological indicators of ecosystem health. Several *Spiranthes* are of conservation concern, with all but four North American taxa listed at the state level and four federally listed species. Since 2017, six species have been described or resurrected, all of which have restricted geographic ranges and urgent conservation needs (Pace and Cameron, 2017). One taxon, *S. bightensis* M. C. Pace ‘Chadds Ford’ (formerly also referred to as a selection of *S. cernua* (L.) Rich. or *S. odorata* (Nutt.) Lindl.), has been used broadly in horticulture, but other species have been largely ignored from this perspective (Pace, 2021). *Spiranthes ochroleuca* (Rydb.) Rydb. is a widespread species native to the northeastern United States that occurs in a wide variety of ecosystem and habitat types ranging from forested edges to highly disturbed sites such as roadsides (Figure 1A). It is relatively common throughout its range and regularly flowers and sets seeds, both indicators that it can serve as a useful model species for determining baseline asymbiotic in vitro germination, seedling development, and ex vitro seedling acclimatization methods for *Spiranthes*.

**Figure 1:**
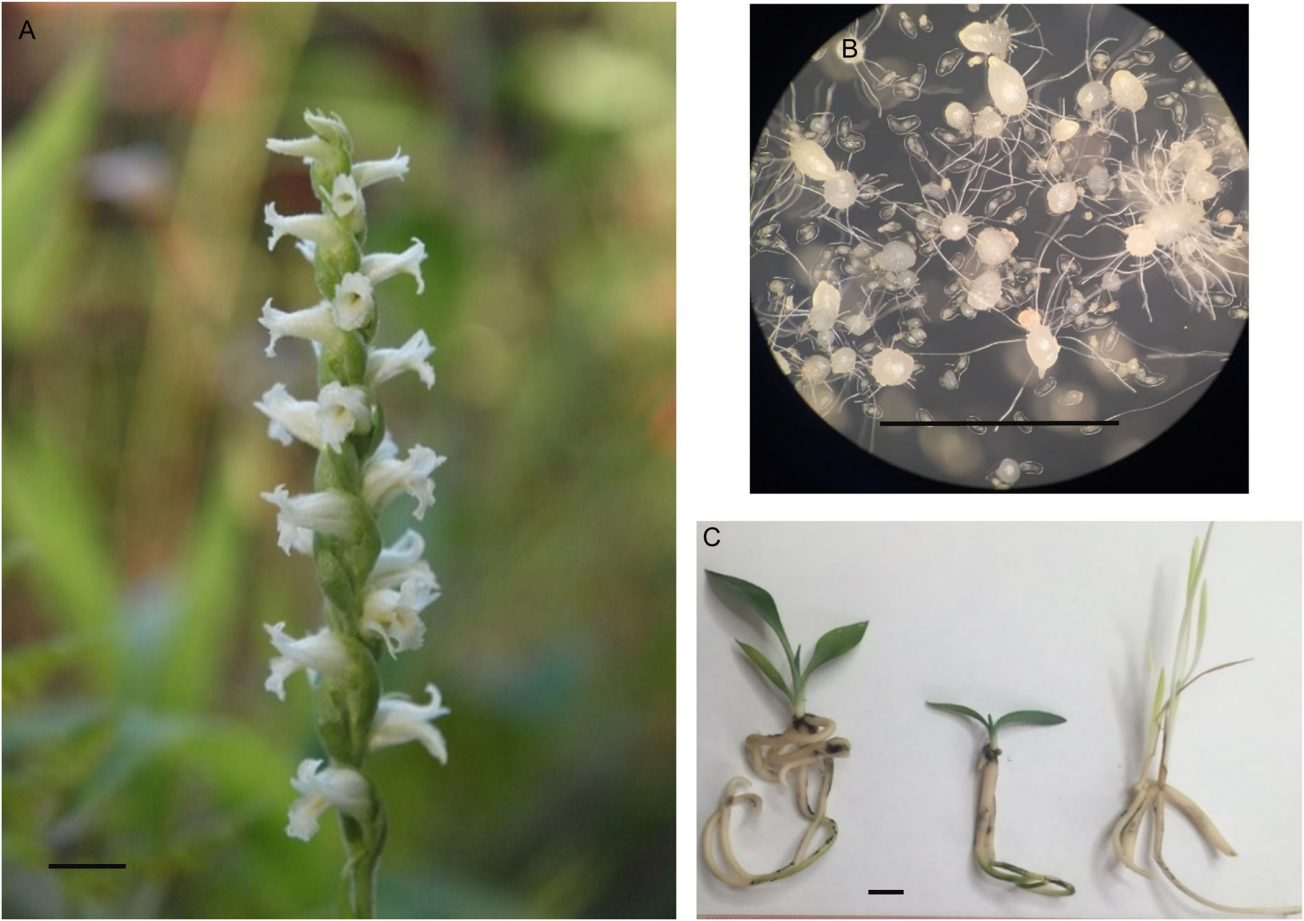
Images of *Spiranthes ochroleuca* from different phases of this study. (A) Flowering plant of *S. ochroleuca* in Elk County, Pennsylvania, September 2017. (B) *Spiranthes ochroleuca* seedlings (32× view) at various stages of development. (C) Seedlings of *S. ochroleuca* from the 24/0‐h L/D (left), 16/8‐h L/D (center), and 0/24‐h L/D (right) photoperiod treatments. The scale bar is 1 cm.

The choice of reagents and timing of orchid seed sterilization in preparation for in vitro culture are two of the major considerations affecting the ability of orchid seeds to germinate. In addition to surface sterilization, the use of reagents also results in scarification of the seed coats through chemical degradation that influences the ability of seeds to imbibe and germinate (Rasmussen, 1995). For *Cypripedium*, there was a direct correlation between the duration of the sterilization/scarification time and an increase in germination percentage, up to the point where the scarifying reagent began to damage seed embryos (Steele, 1996). Seed sterilization and scarification reagents and times often vary widely within and between species and genera of orchids (Rasmussen, 1995). Reported surface sterilization protocols for North American *Spiranthes* are variable, but general recommendations indicate that much shorter times are required for successful germination. Zettler and McInnis (1993) and Stewart et al. (2003) reported 3‐min and 1‐min surface sterilization times for *S. cernua* and *S. brevilabris* Lindl. using 5% NaOCl. Other studies report longer times using different reagents. For *S. delitescens* Sheviak, Hicks (2007) reported successful germination after 16 min using dichloroisocyanuric acid at 5100 ppm. However, Anderson (1990) achieved germination of *S. ochroleuca* and *S. ovalis* Lindl. var. *erostellata* Catling when seeds were sterilized in 7.5% Ca(OCl)_2_ for 20 min, indicating that there are differences in sterilization times depending upon the sterilizing agent used and among taxa. For most North American *Spiranthes* species, there are no reports of seed sterilization times, but existing evidence indicates that the use of NaOCl or Ca(OCl)_2_ requires shortened times for successful in vitro germination.

In vitro germination and seedling development of temperate terrestrial orchids are generally accomplished using either symbiotic or asymbiotic germination protocols. For symbiotic germination, a known orchid mycorrhizal fungus (OMF) is used to stimulate germination, but requires the extra step of fungal isolation and cultivation; for asymbiotic germination, a variety of different culture media are used in absence of a fungus, offering a more simplistic method of propagation that can often yield larger plants in comparison with symbiotic germination (Bustam et al., 2014). Asymbiotic germination is also the standard practice used in the commercial orchid propagation industry. While there are multiple reports of successful asymbiotic germination of *Spiranthes* species, these exist for only a few North American taxa and utilized a small number of existing culture media: water agar, Knudson C, Norstog, Hyponex, Thompson media supplemented with ammonium, Curtis solution 5, and full‐strength Curtis solution (Stoutamire, 1964, 1974; Henrich et al., 1981; Oliva and Arditti, 1984; Anderson, 1991; Hicks, 2007). The wide variety of culture media that have been used to promote in vitro germination and growth of *Spiranthes* in past studies suggests that they are adaptable to various media formulations and that further experimentation is warranted.

There are fewer studies, however, examining the factors that affect the cultivation of temperate terrestrial orchids to maturity after the stages of germination and initial seedling development. These include the necessary photoperiod requirements for continued, proper seedling development and the substrates that promote successful acclimatization of seedlings to ex vitro conditions. Although these factors may influence successful cultivation of orchids as much as the initial germination phases, there are few reports that consider them all in a single study (Bustam et al., 2014).

The variation and gaps in the currently available in vitro and ex vitro *Spiranthes* propagation and cultivation information indicate that development of a baseline, generalizable propagation protocol would be of value. The objectives of this study were to: (1) determine seed scarification/sterilization and culture media that support in vitro, asymbiotic seed germination of *S. ochroleuca*; (2) determine preliminary photoperiod requirements for continued seedling development of *S. ochroleuca*; (3) determine appropriate substrates for ex vitro acclimatization of seedlings; and (4) determine if protocols developed during these studies can be routinely applied to additional seed accessions of *Spiranthes*.

## METHODS AND RESULTS

### Seed source and sterilization

Mature seed capsules of *S. ochroleuca* were collected from six individuals growing at Elk State Forest (Elk and McKean counties, Pennsylvania) and air‐dried for two weeks. Seeds were then transferred to glass vials for cold storage in continual darkness at 3°C and 30% relative humidity (Appendix 1). In preparation for seed germination, seeds were surface sterilized and scarified for 3‐min and 10‐min intervals in a 10% solution of deionized distilled water and household bleach (Clorox; active ingredient 7.4% NaOCl) with two drops of Tween 20. Solutions containing seeds were agitated during both time intervals, and vessels containing seeds were transferred to a sterile working space, removed from the sterilization solution, and rinsed three times in sterile deionized distilled water before plating. Seeds of related taxa were given the same treatment.

### Asymbiotic in vitro seed germination and seedling development

Three asymbiotic orchid seed germination media were examined for their effectiveness in promoting germination and subsequent protocorm development of *S. ochroleuca* seeds. For germination, commercially prepared M551, P723, and Knudson C (K400) (PhytoTech Labs, Lenexa, Kansas, USA) were prepared by autoclaving at 103.4 kPa for 20 min at 122°C. Surface‐sterilized seeds (50–100 per plate) were inoculated onto the surface of sterile germination medium contained in 6‐cm‐diameter Petri plates (ca. 12.5 mL medium/plate; Thomas Scientific, Swedesboro, New Jersey, USA) using a sterile microspatula. Plates were then sealed with Parafilm M (Bemis Company, Neenah, Wisconsin, USA) and incubated under a 0/24‐h light/dark (L/D) photoperiod at 25°C ± 3°C (Appendix 1). Five replicate plates were used for each germination medium treatment, and germination percentage was recorded for a subset of 50 seeds per plate for four to 20 weeks after initiation of the cultures. Seeds were assessed for germination at 4‐wk intervals and classed into one of six categories based on stage of germination and seedling development (Table 1). After reaching stages 3 and 4 at 20 to 28 weeks after sowing, 94 seedlings were transferred to individual 25 × 150‐mm test tubes (Kimble Bomex Labware Group, Beijing, China) with 13 mL of P723. Each was given one of the three photoperiod treatments for 10 months: 24/0‐h L/D, 18/6‐h L/D, and 0/24‐h L/D using T5 fluorescent lights (Hydrofarm, Petaluma, California, USA). Seedlings were then removed from the test tubes and the medium gently scrubbed from the roots. Seedlings were placed in plastic bags with 5 mL of distilled water and placed in a cooler at 1°C ± 2°C for 100 days to satisfy vernalization requirements.

**Table 1:** The six seed and seedling developmental stages of *Spiranthes ochroleuca* used to measure germination and seedling development. Adapted from Stewart and Kane (2007).

Stage	Description
0	No germination, viable embryo
1	Embryo swollen, rhizoids forming (=germination)
2	Continued embryo enlargement, numerous rhizoids
3	Appearance of protomeristem
4	Emergence of first leaf and root initiation
5	Development of second leaf and root development

### Greenhouse acclimatization and substrate screen

Eighty‐three seedlings were then planted in containers in one of four different substrates: pure New Zealand sphagnum (NZ; “New Zealand moss” of horticulture; *Sphagnum cristatum* harvested from the southwestern coast of the South Island of New Zealand and air‐dried; Besgrow Co., Christchurch, New Zealand), Longwood Gardens “Research Mix” (LWG; a peat, composted pine bark, and sterilized soil blend), Good Earth BC5 (GE; a mix of composted pine bark and peanut hulls; Good Earth Horticulture, Edenton, North Carolina, USA), and Sunshine Mix #4 (SS; peat‐based blend; Sun Gro Horticulture, Agawam, Massachusetts, USA) (Appendix 1). Containers were arranged in a randomized, complete block design. Twelve‐ to 14‐month‐old seedlings were placed on a greenhouse table at Longwood Gardens (Kennett Square, Pennsylvania, USA) under 50% shade cloth. While in active growth, seedlings were watered every three to five days as needed and were not fertilized. The number of leaves and leaf length were recorded monthly during the growing season, and the incidence of flowering was also recorded. At the end of the study, the seedlings were harvested from the containers and the fresh weight, number of roots, mean root length, diameter of the longest root, and number of rosettes were recorded.

### Reproducibility of the protocol with other *Spiranthes*

To test the reproducibility of seed germination and seedling development treatments used in this study, we applied the protocol to 11 seed collections of eight additional taxa of eastern North American *Spiranthes* (Table 3).

### Statistical tests

Data were analyzed using the general linear model procedure of SAS statistical software version 9.4 (SAS Institute, Cary, North Carolina, USA). Analysis of variance and means comparison were made with the Waller–Duncan test at *P* = 0.05. Germination data were arcsine transformed to normalize variation.

### Results

**Figure 2:**
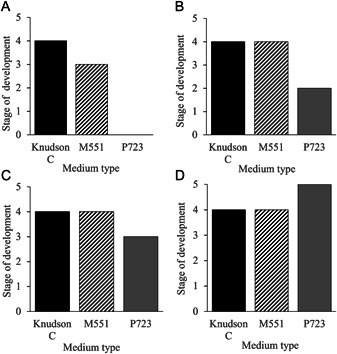
Stages and rates of seedling development of *Spiranthes ochroleuca* on three different culture media over a 12‐week period. (A) Eight weeks of incubation. (B) Twelve weeks of incubation. (C) Sixteen weeks of incubation. (D) Twenty weeks of incubation.

Only seeds from the 3‐min sterilization treatment germinated; embryos of seeds from the 10‐min treatment were all damaged and/or dead upon visual inspection. Seeds from the 3‐min treatments began to swell and germinate on all three media at eight weeks (Figure 2A). Between weeks eight and 20, different stages of germination were apparent on all three media, but seedling development rates varied between media over time (Figure 2). Initial germination was fastest on M551 and Knudson C, but the rate of germination leveled off after 12 weeks and none of the seedlings growing on these media reached stage 5 (Figure 2B, C). Germination on P723 was initially slow, but by week 20 seedling development and overall germination was greater than on the other two media, with several seedlings having reached stage 4 and some even reaching stage 5 (Figure 2D).

For the 3‐min sterilization time, germination occurred on all three media and ranged from 0% to 90%. Germination in P723 was significantly greater than the other two media and ranged from 46% to 90% across treatments, with a mean week 20 germination percentage of 76.8%. Germination on M551 ranged from 0% to 52% across replicates, with a week 20 average of 27.6%. Germination on Knudson C ranged from 0% to 40%, with a week 20 mean of 15.6%. Germination and advanced seedling development were highest and most consistent across replicates on P723.

Seedlings continued to develop in all three photoperiod treatments, but there were significant differences between those grown in complete darkness and those in light treatments. Seedlings incubated on P723 in the 24/0‐h L/D and 16/8‐h L/D treatments had a significantly greater fresh mass, number of roots, and root length than seedlings in other treatments (Table 2). Seedlings in the dark treatment continued to develop, but emerging shoots and leaves showed signs of etiolation (Figure 1C). There were significant differences between the four different substrates in survival. The highest survival rate (95%) and incidence of flowering (20%) occurred on NZ, whereas the lowest survival rate (55%) and incidence of flowering (10%) was on LWG. All media supported seedling survival, indicating that *Spiranthes* are adaptable to a variety of substrates used to support plant growth in greenhouse and nursery environments.

**Table 2:** The effect of three photoperiod treatments on the fresh weight, number of leaves, mean leaf length, number of roots, and root length of *Spiranthes ochroleuca* seedlings.^a^

Photoperiod treatment	Fresh weight (g)	Leaves (No.)	Mean leaf length (mm)	Roots (no.)	Mean root length (mm)
24/0‐h L/D	0.68^a^	2.90^b^	9.25^b^	3.47^a^	14.74^a,b^
16/8‐h L/D	0.66^a^	3.34^a^	6.07^c^	2.93^b^	16.67^a^
0/24‐h L/D	0.35^b^	2.57^b^	17.52^a^	2.67^b^	12.21^b^

^a^ Letters following values represent separation within columns by Waller–Duncan at *P* = 0.05. Numbers followed by the same letter are not significantly different.

The propagation procedures described for *S. ochroleuca* were successful in promoting germination, seedling development, and ex vitro seedling acclimatization in 10 of 11 seed accessions (Table 3). Germination rates and seedling development differed among taxa (data not presented). This suggests that the baseline protocol described for *S. ochroleuca* is broadly applicable to other North American *Spiranthes* taxa.

**Table 3:** In vitro germination, seedling development, and ex vitro acclimatization for 11 seed accessions of *Spiranthes* species using the same methods described for *S. ochroleuca*.

Taxon	Source	Germination on P723	Seedling development (16/8 photoperiod)	Ex vitro establishment
*S. arcisepala* M. C. Pace	Tioga Co., Pennsylvania	X	X	X
*S. bightensis* M. C. Pace	New Castle Co., Delaware	X	X	X
*S. brevilabris* Lindl.	Grimes Co., Texas	X	X	
*S. casei* Catling & Cruise var. *casei*	McKean Co., Pennsylvania	X		
*S. cernua* (L.) Rich.	York Co., Pennsylvania	X	X	
*S. cernua*	Henderson Co., North Carolina			
*S. cernua*	Brazos Co., Texas	X	X	X
*S. cernua* peloric form	Brazos Co., Texas	X	X	X
*S. incurva* (Jenn.) M. C. Pace	Tioga Co., Pennsylvania	X	X	X
*S. lacera* (Raf.) Raf. var. *gracilis* (Bigelow) Luer	Chester Co., Pennsylvania	X	X	X
*S. lacera* var. *lacera* (syn. *S. eatonii* Ames ex P. M. Br.)	Jasper Co., Texas	X		

## CONCLUSIONS

This study demonstrates preliminary seed propagation, in vitro seedling development, and greenhouse acclimatization procedures for North American *Spiranthes*. The following can be concluded: (1) shortened seed sterilization and scarification times are required for successful germination if using sodium hypochlorite; (2) light promotes in vitro seedling development; (3) *Spiranthes* seedlings can be successfully acclimatized to ex vitro conditions on various substrates; and (4) methods successful for propagating *S. ochroleuca* can be successfully applied to other *Spiranthes* taxa, indicating that interspecies variation in germination requirements may be limited and providing key information for working with these exceptional species.

Shortened scarification times are one of the key factors affecting seed germination in all *Spiranthes* species tested for this study. The results presented here indicate that both the integument and testa are rapidly degraded in sodium hypochlorite solutions and the extended bleaching times recommended for other temperate terrestrial orchids will result in seed death for *S. ochroleuca* and related species. This sensitivity to sodium hypochlorite may be attributable to various factors; unfortunately, there is little information available to elucidate these in *Spiranthes*. Thin seed coats, the presence of air space in the integument and between the integument and the embryo, the structural and chemical composition of the integument and seed coat, the presence of different cuticular substances, and “wettability” have all been cited as factors influencing the response of orchid seed coats to chemical reagents (Molvray and Kores, 1995; Arditti and Ghani, 2000; Deconninck and Gerakis, 2021). One issue with such a shortened scarification period using sodium hypochlorite is that bacterial and fungal spores may not be completely killed during the process, leading to contamination of cultures before germination and initial seedling development is complete. Recent research has shown that soaking seeds in a sucrose solution before chemical scarification can cause bacterial and fungal spores to germinate, making them more sensitive to the chemical regent and thereby increasing the efficacy of the sterilant, but this has not been tested with North American *Spiranthes* (Deconninck and Gerakis, 2021). Other scarification reagents should be tested, and alternative options such as ultrasonic treatment after an initial chemical sterilization or culture of immature embryos should be attempted to diversify the range of techniques for successfully germinating *Spiranthes* seeds (Miyoshi and Mii, 1988).

Although all tested media resulted in germination of *S. ochroleuca*, a limitation of this study was the small number of media types used during the germination phase of the experiments. However, our results demonstrate and confirm that germination can occur on culture media with both organic and inorganic nitrogen sources. It is worth noting that *Spiranthes* germinated in the presence of inorganic nitrogen (ammonium nitrate), as it is known to inhibit germination in other temperate terrestrial orchid genera (Rasmussen, 1995; Malmgren, 1996). This suggests that a variety of media with differing nitrogen sources can be used to germinate seeds of *Spiranthes*. In addition to nitrogen source, carbohydrate sources can also play a role in germination and seedling development. None of the tested media contained any type of growth regulator or complex organic additive that have been used to aid germination in other terrestrial orchid genera, indicating that this is an area for experimentation that could be explored, especially as it could impact germination rate and rate of seedling development. There is still room for experimentation, but our results confirm that P723 can be used as a baseline culture media formulation to propagate a broad array of North American *Spiranthes*.

This study determined baseline parameters regarding in vitro germination requirements, light and medium requirements for seedling growth, and preliminary information on ex vitro cultivation requirements of 12 North American *Spiranthes* taxa. This information is broadly applicable to those wishing to initiate *Spiranthes* propagation projects and would be worth testing in other closely related, spiranthoid orchid taxa.

## AUTHOR CONTRIBUTIONS

P.J.Z. and M.T. conceived and designed the experiments. A.C. and P.J.Z. performed all of the experiments. P.J.Z. and J.N. wrote the initial manuscript. All authors approved the final version of the manuscript.

## Supporting information


**Appendix S1**. Data sets providing (1) raw data for asymbiotic seed germination of *Spiranthes ochroleuca*, (2) raw and mean data set of photoperiod effect on seedling development, and (3) raw greenhouse and substrate screen.

## Data Availability

The data sets generated during and/or analyzed during the current study are available in the Supporting Information as Appendix S1.

## APPENDIX 1 Recommended protocol and materials for seed collection, in vitro seed propagation, and ex situ acclimatization of orchid seedlings.

### A.1 Seed collection

Materials required:

1. Fine forceps
2. Hand lens (10×)
3. Bonsai shears
4. Glassine envelopes
5. Glass 1.5‐mL tubes

### A.2 Methodology:

1. Seeds of most eastern North American *Spiranthes* mature at 4 to 6 weeks after anthesis. The capsules are small (ca. 5–7 mm × 2–4 mm), sessile, and produced on a central floral scape. Capsules at the bottom of the scape will mature and dehisce before capsules at the apex.
2. Before collection, use the hand lens to check for the presence of viable seeds. Viable seeds will have an apparent proembryo in the center of the seed, whereas non‐viable seeds will usually present as an empty testa lacking the proembryo.
3. Seeds can be collected in one of two ways depending on the level of maturity. If capsules have split and seeds are dehiscing, seeds can be tapped into a glassine envelope until the necessary amount is obtained. If the seeds have not yet dehisced, individual capsules can be removed using fine forceps or bonsai shears and placed in glassine envelopes. Capsules at the bottom of the spike should be collected first. In some cases, it may be possible or necessary to collect the entire floral spike, in which case it should be severed near the base of the inflorescence.
4. Seeds are then air‐dried for a period of 10 to 14 days after collection.
5. After the drying process, the seeds can be stored in 1.5‐mL glass tubes until needed for the next step. Seeds should be stored at low temperature and low humidity until in vitro seed sowing takes place.
6. Please note that less than 10% of seeds produced in each population should be collected at any one time.

### A.3 In vitro seed propagation

Materials required:

1. Forceps
2. Inoculating loop
3. Sterile Petri dishes
4. 25 × 150‐mm test tubes
5. Commercial preparations of M551, P723, and K400 (PhytoTech Labs, Lenexa, Kansas, USA)
6. Parafilm (Bemis Company, Neenah, Wisconsin, USA)
7. Autoclave
8. Erlenmeyer flask, 1000 mL
9. Screw‐top glass jar, 1000 mL
10. Hot plate stirrer and stir bar
11. Sterilized paper towels
12. Sterilized Whatman filter papers
13. Sterilized glass funnels
14. Freshly prepared 10% hypochlorite solution
15. 70% ETOH
16. Gloves for handling hot materials

### A.4 Procedure:

1. Select seed germination medium of choice, mix with water as directed in a 1000‐mL Erlenmeyer flask, and bring to a boil on the hot plate stirrer. Once the medium is dissolved, transfer to a 1000‐mL screw‐top glass bottle and autoclave at 104°C at 15 psi for 25 min.
2. While the medium is in the autoclave, place sterile Petri dishes (with the lids off) in the sterilized laminar flow hood or otherwise sterile working space.
3. Once the autoclave process is completed, put on the gloves and remove the hot medium from the autoclave. Pour 10–15 mL of hot medium into each Petri dish and allow to cool before covering. Once the medium has cooled and solidified, the plates can be used immediately, or covered and wrapped in Parafilm to be used later.
4. To prepare for seed sowing, remove the seeds from the cooler and allow them to acclimate to room temperature conditions.
5. Once seeds have reached room temperature, the desired quantity can be removed from the vessel for processing.
6. Place aliquots of seeds in glass vials containing the hypochlorite solution for sterilization and scarification for a maximum of 3 min. Please note that this time varies for other orchid genera and is exceptionally short for *Spiranthes*.
7. In the laminar flow hood, remove the seeds from the sterilizing solution by pouring them over sterilized filter papers placed in a sterilized filter funnel. Seeds are then rinsed three times with sterile water and allowed to air‐dry.
8. Once the seeds have dried, inoculate them onto the medium in the Petri dishes using an inoculating loop. Seeds can also be tapped onto the medium.
9. To ensure an even distribution of seeds for counting, place the seeds into drops of sterile water placed on the medium and use the inoculating loop to distribute them over the plate.
10. Once the seeds are in place, seal the plates with Parafilm and place in dark conditions at room temperature. The plates should be monitored for contamination every few days during the first two weeks after sowing.
11. Once the seeds begin to germinate and develop to stage 5 or 6 (Table 1), the seedlings can be transferred to fresh medium for continued growth.
12. To accomplish this, surface sterilize the Petri dishes containing seedlings using 70% ETOH and place in the sterilized laminar flow hood. Then open the dishes, carefully remove the seedlings from the Petri dish using sterilized forceps, and place into test tubes containing at least 13 mL of medium.
13. Once the seedlings have been transferred to the test tubes, seal the test tubes with Parafilm and place the test tube racks in a lighted environment for continued growth and development.

### A.5 Ex vitro acclimatization

Materials required:

1. Large forceps
2. Soft wire brush or toothbrush
3. Colander
4. Black, 8.9 cm wide × 8.9 cm deep, square plastic containers (HC Companies, Twinsburg, Ohio, USA)
5. Condensed bale of New Zealand sphagnum
6. Good Earth BC5 potting mix (Good Earth Horticulture, Edenton, North Carolina, USA)
7. Sunshine Mix #4 (Sun Gro Horticulture, Agawam, Massachusetts, USA)
8. Access to water and controlled growth environment

Procedure:

1. Once the seedlings have reached a size where they can withstand transplanting to ex vitro conditions, they can be removed from the test tubes.
2. Carefully remove the seedlings from the test tubes using large forceps, place in a colander, and rinse with tepid water to remove the tissue culture medium.
3. All the media should be removed from the roots of the orchid seedlings before transplanting. This may require use of a brush to gently remove media from hard‐to‐reach areas. Orchid roots are extremely brittle at this stage and should be handled with care to prevent abrasions to or breaking of the roots.
4. Place seedlings in a plastic bag or in damp paper towels to prevent desiccation until planting.
5. To prepare for planting, select the potting medium of choice and fill containers to within 4 cm of the top.
6. Place one seedling in the container and backfill with potting medium to within 1.5 cm of the top. Gently firm in the medium using your fingers, being careful not to compress to the point where the air‐filled pores in the medium are reduced.
7. Once the seedlings are potted, carefully water them and place on the greenhouse bench or nursery environment under 50% shading. In our experience, *Spiranthes* seedlings do not require a hardening off period under mist like other some other types of orchids after removal from in vitro conditions.
